# A Novel Approach for Measurement of Composition and Temperature of N-Decane/Butanol Blends Using Two-Color Laser-Induced Fluorescence of Nile Red

**DOI:** 10.3390/s20195721

**Published:** 2020-10-08

**Authors:** Matthias Koegl, Mohammad Pahlevani, Lars Zigan

**Affiliations:** 1 Lehrstuhl für Technische Thermodynamik (LTT), Friedrich-Alexander-Universität Erlangen-Nürnberg (FAU), 91058 Erlangen, Germany; Mohammad.pahlevani@fau.de (M.P.); lars.zigan@fau.de (L.Z.); 2 Erlangen Graduate School in Advanced Optical Technologies (SAOT), Friedrich-Alexander-Universität Erlangen-Nürnberg (FAU), 91052 Erlangen, Germany

**Keywords:** absorption, LIF, two-color detection, butanol admixture, blend

## Abstract

In this work, the possibility of using a two-color LIF (laser-induced fluorescence) approach for fuel composition and temperature measurements using nile red dissolved in n-decane/butanol blends is investigated. The studies were conducted in a specially designed micro cell enabling the detection of the spectral LIF intensities over a wide range of temperatures (283–423 K) and butanol concentrations (0–100 vol.%) in mixtures with n-decane. Furthermore, absorption spectra were analyzed for these fuel mixtures. At constant temperature, the absorption and LIF signals exhibit a large spectral shift toward higher wavelengths with increasing butanol concentration. Based on this fact, a two-color detection approach is proposed that enables the determination of the butanol concentration. This is reasonable when temperature changes and evaporation effects accompanied with dye enrichment can be neglected. For n-decane, no spectral shift and broadening of the spectrum are observed for various temperatures. However, for butanol admixture, two-color thermometry is possible as long as the dye and butanol concentrations are kept constant. For example, the LIF spectrum shows a distinct broadening for B20 (i.e., 80 vol.% n-decane, 20 vol.% butanol) and a shift of the peak toward lower wavelengths of about 40 nm for temperature variations of 140 K.

## 1. Introduction

The usage of renewable fuels such as ethanol and butanol can contribute to a reduction of CO_2_ emissions. Modern direct-injection spark-ignition (DISI) engines, which are commonly used in passenger cars, have a higher fuel efficiency in comparison to conventional port fuel injection (PFI) engines. The fuel atomization (forming a spray of small droplets) and evaporation controls the subsequent process steps including mixture formation, ignition, heat release, pollutant formation, and finally system efficiency. This process chain is intricate, and renewable fuels and fuel blending additionally increase the complexity of the spray and mixture formation processes. Overall, physical fuel properties govern atomization, evaporation, and mixture formation. Especially ethanol as a biofuel with vastly different properties needs special attention as current DISI engines are operated with ethanol admixture up to 20 vol.% to gasoline, while E85 (i.e., 85 vol.% ethanol in gasoline) is common in some countries as well [1]. Butanol is another promising biofuel candidate for gasoline [2,3,4], diesel [5,6], and kerosene fuels [7]. It shows a higher boiling point and a much lower evaporation enthalpy than ethanol, which could lead to improved combustion behavior under cold start conditions (under which ethanol may show increased pollutant emissions) [3]. 

Atomization as an essential process step can be characterized by, e.g., using planar droplet sizing (PDS). With this imaging technique, which was proposed over two decades ago [8], a map of droplet sizes can be determined in form of the Sauter mean diameter (SMD). It is based on the ratio of the laser-induced fluorescence (LIF) signal of the liquid phase and the Mie-scattering signal governed by the surface of droplets. For this approach, the fuel is usually doped with a suitable tracer or dye. The LIF/Mie technique is based on the *d^3^*-dependence of the LIF signal and the *d^2^*-dependence of the Mie-signal of laser-illuminated droplets [9,10,11,12,13]. The qualitative LIF/Mie ratio can be calibrated by PDA measurements or with a droplet generator [2,13,14,15]. Fuels that contain aromatic components such as toluene, benzene, or naphthalene showing absorption bands in the UV-region can be used without tracers for creating an LIF signal [16]. However, aromatic hydrocarbons are rarely used without the addition of suitable tracers, since they show a high temperature and oxygen sensitivity, which can lead to large systematic errors [17,18]. While many studies in the literature deal with alcohol and water sprays [2,15,19,20], only a few studies deal with realistic fuels such as gasoline and diesel [21,22]. 

The well-characterized fluorescence dye Eosin Y was utilized in earlier PDS studies [2,15,20,23,24,25] in ethanol and butanol sprays, but it is not soluble in alkanes. The tracer nile red (C_20_H_18_N_2_O_2_) is commonly applied in the field of microfluidic systems and biology [26,27] and is also a suitable tracer for alkanes [28,29]. Nile red is a suitable tracer for PDS in alkanes due to its low temperature dependency at moderate ambient conditions. The temperature dependency of the dye dissolved in the gasoline surrogate fuel Toliso (consisting of 65 vol.% iso-octane and 35 vol.% toluene) was already reported in [29]. The accuracy of the LIF/Mie ratio technique for droplet sizing depends on many boundary conditions. Partial fuel evaporation leads to an increase of the dye concentration in the shrinking droplet. This results in an increase of the LIF/Mie ratio and an overestimation of the droplet size. Droplet heating and evaporation lead to variation of the droplet temperature. While the Mie scattering signal shows a low temperature dependence, the LIF signal is usually more temperature dependent, so a variation of the temperature results in an over- or underestimation, respectively, of the droplet size. This behavior has already been investigated in previous studies of the authors, dealing with ethanol [15] (tracer Eosin Y) and E20 [30] (80 vol.% Toliso, 20 vol.% ethanol; tracer nile red). The investigated temperature dependence of the LIF signal at moderate temperatures is in the range of 7% (ethanol, Eosin Y, temperature interval: 296–324 K) and 13% (E20, nile red, temperature interval: 263–293 K) [29]. However, the temperature-dependent LIF signal can be used for thermometry in form of a two-color approach to correct for temperature effects on the LIF signal. Furthermore, the fluorescence spectrum depends on the solvent. For example, a 20 vol.% admixture of ethanol to Toliso leads to a spectral shift of 50 nm toward higher wavelengths [29]. For butanol mixed with other reference or surrogate fuels, there is no detailed investigation of different blends on the fluorescence emission. In case of a spectral variation of the fluorescence with varying mixture composition, this behavior could be utilized to determine the butanol concentration, e.g., in evaporating gasoline, diesel, or kerosene droplets. However, for other dyes such as Eosin Y or rhodamines, the emission spectra are similar when the dyes are dissolved in ethanol or butanol, respectively [31].

The present study focuses on the determination of temperature and butanol concentration in a sample by using the spectral fluorescence emission of nile red doped to various fuel mixtures. For this purpose, absorption and emission measurements of the dye in n-decane/butanol mixtures are conducted as a function of butanol content to evaluate its applicability for thermometry and concentration measurements. First, the absorption and emission spectra are investigated as a function of butanol concentration. Second, suitable filters for a determination of the butanol concentration are suggested and validated. Third, the temperature-dependent emission is measured and suitable filters for thermometry are suggested and validated. Finally, a brief discussion of the 2-color techniques is provided.

## 2. Description of the Experiment

### 2.1. Experimental Setup: Fluorescence Spectroscopy

**Figure 1:**
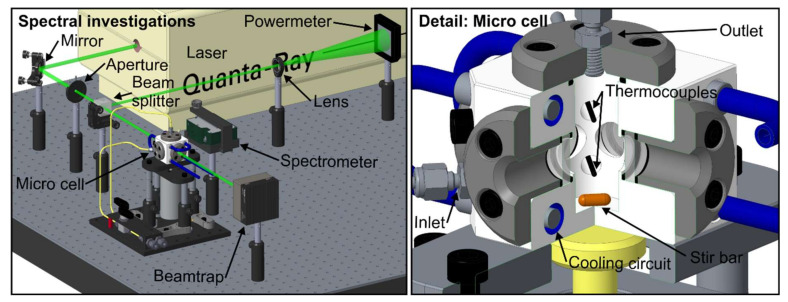
Optical setup (**left**) and internal design (sectional view) of the micro cell (**right**).

The optical setup and a sectional view of the micro cell are shown in Figure 1. The probe volume within the micro cell is illuminated by a pulsed Nd:YAG laser (model 150-10, Quanta Physics, USA; wavelength 532 nm, repetition rate 10 Hz, pulse width >10 ns). An electrically attenuated shutter inside the laser enables the illumination of the probe only during the actual LIF measurements. This procedure also avoids possible photo-dissociation effects due to constant probe illumination. The emitted laser beam cross-section is cut down to 4.2 mm by an aperture. Then, the beam is bisected by a beam splitter to enable simultaneous monitoring of the laser fluence (power meter: model QE50LP-S-MB-INT-D0, Gentec Electro-Optics, Canada) during the measurements. One of the bisected beams passes the measurement volume in the micro cell (which was used for temperature-dependent measurements) or a cuvette (used for concentration studies), respectively. A spectrometer (model USB 4000, Ocean Optics, USA, wavelength range 495.9–831.8 nm, 3648 pixels, slit size 10 µm, integration time 100 ms, 50 subsequent spectra were averaged for each measurement) records the LIF spectra under a detection angle of 90°. For studying the effect of dye concentration and butanol concentration on the fluorescence signal, which was conducted at ambient conditions (0.1 MPa, 293 K), a cuvette with a quadratic cross-section (Hellma analytics, edge length 10 mm, 3.5 mL) was used. The temperature-dependent emission spectra were recorded in the micro cell. The micro cell features four optical accesses (½" sapphire windows, optical access diameter: 9 mm, inner distance between two windows: 19.1 mm). A built-in magnetic stirrer (stir bar: 8 mm × 3 mm, 1500 rpm) ensures a homogeneous temperature distribution within the cell. The temperature within the micro cell is monitored by two thermocouples (type K, tc-direct GmbH, Germany). Cooling/heating circuits in the cell body in combination with a recirculating thermostat (model: FP50, Julabo, Germany) enable a wide range of investigated temperatures.

### 2.2. Experimental Setup: Absorption Spectroscopy

The tracer and fuel concentration-dependent absorption measurements were performed using an UV/VIS spectrometer (model V-750, JASCO, Japan, light sources: halogen and deuterium lamps, wavelength range 190–900 nm, 3551 pixels, spectral bandwidth 2 nm, scan speed 200 nm/min). A cuvette was used instead of the micro cell as no temperature spectra are recorded. 

## 3. Fuels and Dye Used

Modern DISI engines are operated with gasoline and increasingly with ethanol admixtures up to 20 vol.%, although higher ethanol content in gasoline such as E85 is also common in some countries for PFI IC engines. In a previous study, we studied the effect of multi-component fuels (e.g., kerosene, “Toliso”) and mixtures with biofuels (farnesane, HEFA (Hydroprocessed Esters and Fatty Acids), ethanol) on the absorption and fluorescence behavior of nile red. In the present work, we selected pure n-decane as solvent, which is often applied as surrogate fuel for gasoline. In this study, n-decane was utilized as it is a model fuel representing the upper boiling point range of gasoline (see, e.g., [32]) or the lower boiling point range of kerosene and diesel fuels. 

N-decane is a non-polar molecule (polarity −0.3), which hardly affects the absorption and emission of dyes, while butanol (polarity 3.9) exhibits a higher polarity [33]. 

Nile red (C_20_H_18_N_2_O_2_, Sigma Aldrich) belongs to the group of fluorophores, and its aromatic ring structure features polar substituents. This polar substituents lead to a high sensitivity to the chemical and physical environment of surrounding solvent molecules [34]. For the investigated solutions, dye concentrations of 0.47–30 mg/L were tested. A minimum of 3.5 mg of nile red was weighed with a high-precision analytical scale (Mettler Toledo XS 205, proofed repeatability 0.05 mg). The dye was completely dissolved in all investigated fuel–dye mixtures. The solutions were diluted to adjust the respective dye concentrations. The dye has a melting point of 476–479 K), but this temperature is above the maximum tested temperature in the present micro cell setup. 

## 4. Results

This section is structured as follows. First, the dye concentration effect on the absorption and fluorescence spectra (excitation wavelength: 532 nm) of n-decane and B20 is investigated. Second, the temperature influence on the emission spectra of the investigated fuel/dye mixtures is analyzed. Third, the effect of the butanol content on the absorption and emission spectra is investigated. Finally, a brief discussion of the data is presented. All spectral results are presented in the visible wavelength range (380–780 nm), which is most relevant for the absorption and emission of nile red [29].

### 4.1. Concentration Study

**Figure 2:**
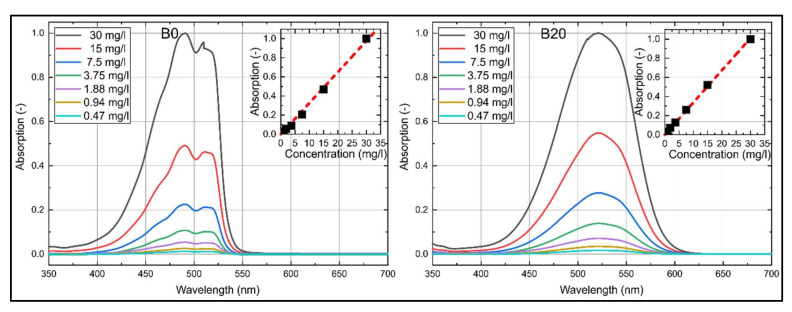
Absorption spectra of nile red (**left**: in n-decane (B0), **right**: in B20) normalized to the respective maximum intensity at 30 mg/L, inserted diagrams show linearity (B0: R^2^ = 0.997, B20: R^2^ = 0.999) of the integral LIF (laser-induced fluorescence) signal for various dye concentrations; 293 K.

The absorption spectra of nile red dissolved in n-decane (B0) and B20 are shown in Figure 2 for various dye concentrations at reference conditions (293 K, 0.1 MPa). For both fuels, nile red shows a distinct absorption between 350 and 600 nm. A butanol admixture of 20 vol.% leads to a shift of the absorption spectrum of roughly 21 nm toward higher wavelengths. The absorption spectra of nile red in n-decane are characterized by two peaks at 490 and 514 nm, while the left peak is more pronounced and more distinct as compared to Toliso [29]. The absorption spectrum of nile red in B20 is characterized by one broad single peak around 521 nm. The absorption intensities for both fuels increase linearly with higher dye concentration for all investigated concentrations. The coefficient of determination *R*^2^ for the linear fit curves displayed in Figure 2 is 0.997 for B0 and 0.999 for B20. 

**Figure 3:**
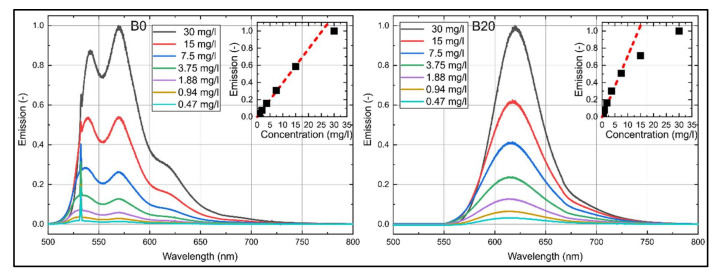
LIF emission spectra of nile red (**left**: in n-decane (B0), **right**: in B20) normalized to the respective maximum intensity at 30 mg/L, inserted diagrams show linearity (B0: R^2^ = 0.999, B20: R^2^ = 0.993) of the integral LIF signal for various dye concentrations; 293 K.

The concentration-dependent emission spectra of nile red in B0 and B20 at reference conditions (293 K, 0.1 MPa) are shown in Figure 3. For both fuels, the dye shows an emission between 500 and 750 nm. A butanol admixture of 20 vol.% leads to a shift of the emission spectra of roughly 65 nm toward higher wavelengths, which is doubled compared to the spectral absorption shift. Similar to the absorption spectra, the emission spectra of B0 are characterized by a double peak, which is again more distinct as compared to Toliso and kerosene [29]. Here, the first peak is at 539 nm, and the second peak at 570 nm. Here, the left peak is more pronounced for all investigated concentrations except for 30 mg/L, where the right peak is more pronounced. The emission spectra of B20 show one single peak at 620 nm. The emission intensities of B0 increase linearly with higher dye concentration up to a dye concentration of 15 mg/L. B20 shows a linear behavior up to a dye concentration of 7.5 mg/L. Beyond this concentration, the distinct absorption of laser light along the beam path takes place so that the intensity increases less. Furthermore, also a part of the emission is re-absorbed by the dye, which is visible in the emission spectrum between 550 and 600 nm. There, the signal increases less with larger dye concentration. The coefficient of determination R^2^ for the linear fitting curves displayed in Figure 3 is 0.999 for B0 (up to 15 mg/L) and 0.993 (up to 7.5 mg/L) for B20. 

**Figure 4:**
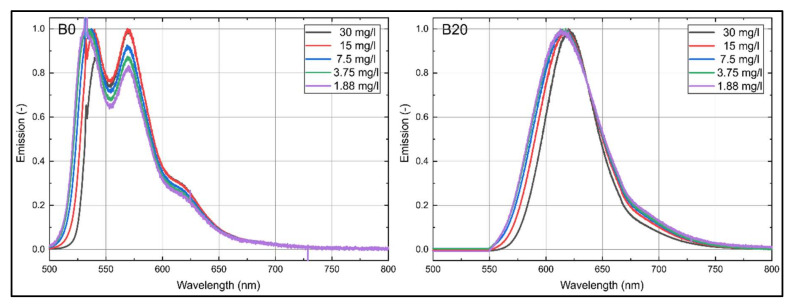
Normalized LIF emission spectra of nile red in B0 and B20 for various dye concentrations; 293 K.

For a closer examination of the small spectral changes induced by concentration variations, the normalized emission spectra of B0 and B20 for various dye concentrations are provided in Figure 4. B0 shows no spectral shift but a change in the ratio of the two peaks. At a high dye concentration (30 mg/L), the right peak is more pronounced. With lower dye concentration, the left peak gets more and more pronounced and dominates the decreasing right peak. Furthermore, the left emission flank exhibits a spectral shift of approximately 10 nm toward higher wavelengths (between 1.88 and 30 mg/L) with increasing dye concentration. B20 shows a similar concentration-dependent behavior. Here, the left emission flank exhibits a spectral shift of approximately 12.5 nm toward higher wavelengths (between 1.88 and 30 mg/L) with increasing dye concentration. 

The concentration-dependent behavior is mainly caused by reabsorption effects and has to be taken into account in quantitative measurements to reduce the influence of systematic errors. This is of high relevance when partial evaporation occurs and when the influence of dye enrichment cannot be neglected (e.g., within shrinking droplets, but this needs further investigation).

### 4.2. Temperature-Dependent Emission Spectra

**Figure 5:**
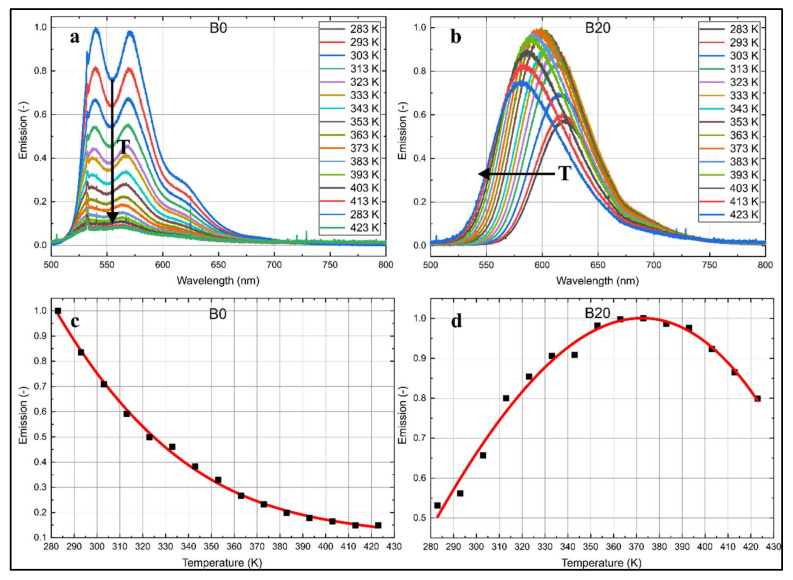
Temperature-dependent normalized emission spectra of nile red (7.5 mg/L) in B0 (**a**) and B20 (**b**) normalized to the individual maximum intensity; normalized integral fluorescence intensities (**c**: B0, **d**: B20) for various temperatures.

The temperature-dependent emission spectra of B0 and B20 are shown in Figure 5 for a wide range of temperatures (283–423 K). B20 shows no distinct peaks, while B0 exhibits two characteristic peaks in the whole temperature range. Nile red dissolved in B0 and B20 shows a distinct temperature dependence of the fluorescence emission. In the case of B0, higher temperatures lead to a decrease of the fluorescence signal (Figure 5c). The signal drops by about 85% when the temperature increases from 283 to 423 K. For B20, at first, higher temperatures cause an increase of the integral fluorescence signal to 188% from 283 up to 373 K; afterwards, the fluorescence decreases again to 150% of the initial intensity at 283 K. It should be noted that this signal drop at very high temperatures is not due to dye dissociation. The measurements were repeated from high temperatures to low temperatures, and the same curves were acquired again. 

**Figure 6:**
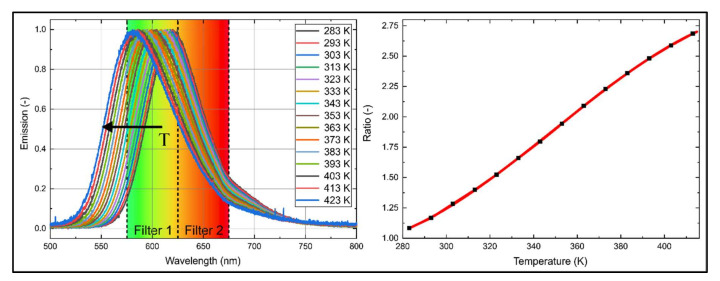
Temperature-dependent emission spectra of nile red (7.5 mg/L) in B20 (**left**) normalized to the maximum intensity and the intensity ratio (R^2^ = 0.999) for two-color thermometry (**right**) for various temperatures.

Nile red dissolved in n-decane exhibits only an intensity reduction in the complete spectrum without spectral shifts for varying temperatures. The emission of nile red dissolved in B20 shows a shift of the left flank toward lower wavelengths, while the right flank stays unchanged. This behavior can be utilized for the determination of the liquid temperature using a two-color detection scheme. The normalized integral fluorescence intensities in Figure 6 visualize the temperature-dependent change of the spectral intensities. 

Two-color thermometry imaging uses the signal ratio in two spectral bands to determine the temperature of a sample. A detection scheme was evaluated using a suitable combination of two band pass filters centered at 600 and 650 nm, respectively (e.g., Edmund optics, #84-785 (600 nm, 50 FWHM), #84-786 (650 nm, 50 FWHM)), is shown for B20 in Figure 6. Since the measurement approach is intended for a later application in a droplet chain or a levitator (where single droplets lead to low signals), relatively broadband filters with 50 FWHM were used to ensure sufficient large fluorescence intensities.

The chosen filter bands are visualized together with the normalized spectrum in Figure 6 (left), and the corresponding signal ratio is presented in Figure 6 (right). The ratio was determined using the individual filtered intensities, which have been deduced from the multiplication of the fluorescence spectra with the respective filter curves. The ratio exhibits approximately a linear behavior with increasing temperature. This filter combination allows a good temperature sensitivity (on average 1.2%/K). However, it should be noted that re-absorption effects in the “left” spectral band could not be excluded.

The temperature can be determined with the ratio *r*_Temperature_ (R^2^ = 0.999) using the following polynomial equation:

(1) $$T(K)=17.29-0.16\cdot r_{{}_{Temperature}}+5.01\cdot r_{{}_{Temperature}}^{{}^{2}}-4.77\cdot r_{{}_{Temperature}}^{3}.$$

The ratio *r_Temperature_* can be determined using the ratio of the two products of the transmission curves *τ* of the respective filters and the fluorescence signal *I_LIF_* (when other efficiencies of the optical setup (e.g., cameras) are neglected):

(2) $$r_{{}_{Temperature}}=\frac{\sum \tau_{Filter\_600nm}\cdot I_{LIF}}{\sum \tau_{Filter\_650nm}\cdot I_{LIF}}$$

### 4.3. Absorption and Emission Spectra of Various Butanol/n-Decane Mixtures

**Figure 7:**
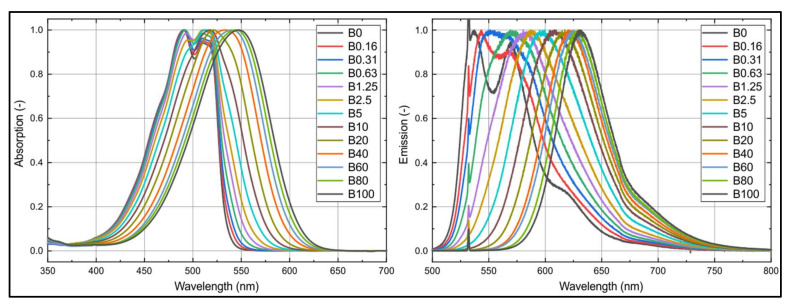
Absorption (**left**) and emission spectra (**right**) of nile red (7.5 mg/L) in n-decane at various butanol concentrations normalized to the respective maximum values; 293 K, 0.1 MPa.

The emission and absorption spectra of nile red in n-decane are presented in Figure 7 for different butanol concentrations. A butanol admixture to n-decane leads to a shift of the absorption and emission spectra toward higher wavelengths. The displacement of the emission spectra is in the range of two times the absorption shift. Similar behavior was also observed in an earlier study for Toliso mixed with ethanol (E20 and E40) [29]. The absorption and emission spectra are very sensitive to butanol admixture, especially at low butanol concentrations (up to 10 vol.%). This sensitivity is reduced with increasing butanol percentage. The two characteristic spectral peaks of n-decane already merge at very low butanol concentrations (i.e., below 0.31 vol.%). 

**Figure 8:**
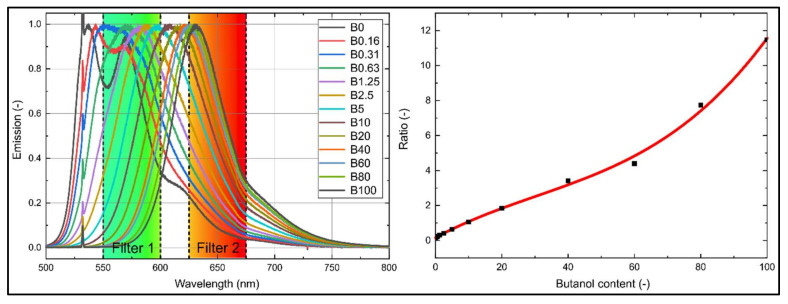
Normalized emission spectra of nile red (7.5 mg/L) in n-decane at various butanol concentrations and an overlay of suitable filters (**left**); intensity ratio (**right**, R^2^ = 0.997); 293 K, 0.1 MPa.

The butanol-dependent spectral shift can be used to determine the composition of the mixture using another intensity ratio technique. A suitable combination includes the two band pass filters 575 and 650 nm (e.g., Edmund optics, #86-952 (575 nm, 50 FWHM), #84-786 (650 nm, 50 FWHM)). The chosen filter bands are overlaid in the normalized fluorescence spectra in Figure 8 (left). The concentration-dependent intensity ratio is provided in Figure 8 (right). The ratio was determined using the individual filtered intensities, which have been deduced from the multiplication of the fluorescence spectra with the respective filter curves. The ratio exhibits a roughly linear behavior with increasing butanol concentration. This filter combination allows for a high butanol concentration sensitivity (on average 9.8%/vol.%, calculated for 20–80% butanol admixture). 

The butanol content of a probe at a temperature of 293 K can be determined by using the ratio *r_Concentration_* (R^2^ = 0.997) with the following polynomial equation:

(3) $$C(vol.\%)=0.15884+0.10254\cdot r_{Concentration}-0.0012\cdot r_{{}_{Concentration}}^{{}^{2}}+1.31418\cdot 10^{-5}\cdot r_{{}_{Concentration}}^{3}$$

with:

(4) $$r_{{}_{Concentration}}=\frac{\sum \tau_{Filter\_575nm}\cdot I_{LIF}}{\sum \tau_{Filter\_650nm}\cdot I_{LIF}}.$$

### 4.4. Discussion of the Data

In the previous sections, a strategy for the measurement of the temperature and butanol concentration was presented for butanol/n-decane mixtures. The focus of the presented work is on the potential application of these techniques in sprays and single droplets. Both techniques for the measurement of the temperature as well as the butanol concentration are only applicable if either the concentration (for thermometry) or the temperature (for composition measurements) is fixed. Systematic measurement errors will occur if both the temperature and the butanol concentration change significantly e.g., during preferential evaporation of one component. Preferential evaporation leads to a variation of the butanol/n-decane ratio. This effect is also known for some iso-octane/ethanol-mixtures [35]. For example, for E20, it is found that ethanol evaporates faster than iso-octane at certain conditions, while for E85, the sequence can be in inverse [35,36,37]. Similar behavior is expected for butanol admixture. The temperature of the droplet may also change during its heat-up and evaporation [38]. The ongoing evaporation will lead to a distinct temperature drop of the ambient gas due to the evaporation enthalpy, especially for large butanol concentrations. Furthermore, the dye concentration will change, but this is less problematic for thermometry in n-decane (and it is more serious for B20, see Figure 4, as the signal ratio will be affected). 

## 5. Conclusions and Future Work

The suitability of the temperature and fuel composition determination based on the laser-induced fluorescence of nile red solved in n-decane and B20 was investigated in a cuvette and a micro-cell, respectively. This proof-of-concept study is the basis for the subsequent development of the technique for application in droplet and spray measurements. The temperature-dependent LIF emission spectra were recorded in a specially designed micro cell in a wide range of temperatures (283–423 K). First, the influence of laser fluence and dye concentration on the linearity of the absorption and LIF signal were studied. The LIF intensity of n-decane shows no temperature-dependent spectral shift at all. Only a distinct signal drop with temperature was observed. For B20 and increasing temperatures, a spectral broadening and shift of the peak LIF intensity toward lower wavelengths was observed, allowing the determination of the temperature in case that the dye and butanol concentration is kept constant. For B20, the selected filter pair for two-color LIF thermometry allows an average temperature sensitivity of 1.2%/K. 

The spectral LIF intensities for varied butanol concentration were recorded from B0 (pure n-decane) to B100 (butanol). With increasing butanol concentration, the emission spectra showed a distinct spectral shift toward higher wavelengths. A selected filter pair allows a butanol concentration-dependent sensitivity of 9.8%/vol.% on average. The measurement technique allows a determination of the butanol concentration in the fuel blends when evaporating effects (leading to dye enrichment) and temperature changes can be neglected. In summary, the two-color LIF approaches allow either a determination of the temperature (when the butanol concentration is constant) or the butanol concentration (when the temperature is kept constant). The technique is applicable for thermometry studies in a confined heated volume or for isothermal mixing of these two fuel components. In general, for measurement in droplets and sprays, the absorption and emission experiments as well as the respective calibration has to be repeated, since the re-absorption in parts of the LIF spectrum is different in comparison to the condition in the cuvette or cell. This will lead to different sensitivities of the intensity ratios.
